# Countering failures of imagination: governance, human development and public health in low- and middle-income countries

**DOI:** 10.1080/16549716.2026.2748104

**Published:** 2026-10-16

**Authors:** Kathryn G. Watt, Dhyan Saravanja, Sean Beckett, Chris Desmond

**Affiliations:** a Centre for Research in Health Systems, School of Nursing and Public Health, University of KwaZulu Natal, Durban, South Africa; b Department of Economics and Finance, University of the Witwatersrand, Gauteng, South Africa; c Health Economics and HIV/AIDS Research Division, University of KwaZulu Natal, Durban, South Africa; d Derek Schrier and Cecily Cameron Chair in Development Economics, Department of Economics and Finance, University of the Witwatersrand, Gauteng, South Africa

**Keywords:** Health policy, health governance, whole of government, social sector, human development

## Abstract

Dramatic cuts in global aid have irrevocably changed the health funding landscape in Low- and Middle- Income Countries (LMICs), creating significant challenges for national governments. But there is a silver lining: these changes give national governments greater agency to set development goals. This moment of disruption demands bold thinking; it is an opportunity to place human development above sector-specific outcomes at the heart of national priority setting and reframe investments in public health accordingly. The social conditions that shape a person’s health are the same conditions that shape their education, wealth, and a myriad of other outcomes within the social sector. Yet, despite strong momentum towards intersectoral collaborative approaches to governance, particularly in health, many government departments across the social sector continue to operate as siloes, neither prioritising nor implementing investments that target common or overlapping causes to improve outcomes across sectors. The dominant models of governance are insufficient to this task. We need innovations in governance that move beyond both traditional sector-specific approaches and newer intersectoral frameworks towards restructured government models that fundamentally transcend sectoral boundaries. We describe the limitations of current approaches, and explore how alternative governance structures might look in practice, and consider their implications for the health sector.

## Paper Context

- **Main findings:** Health, education and welfare share causes; holistic life course investment is key.
- **Added knowledge:** Integrated governance, not coordination, is needed for equitable outcomes.
- **Global health impact for policy and action:** Governance must be restructured around human development goals.

## Background: a holistic life course perspective on human development

Good physical and mental health is valuable in and of itself. Yet good health alone is insufficient to ensure human development. The Covid-19 pandemic and dramatic cuts in global aid revealed the malleability of seemingly incontrovertible approaches to governance. The distribution of power has shifted as national governments in Low- and Middle- Income Countries (LMICs) may determine investment priorities with less consideration of multilateral agencies’ and private funders’ wants. Against emerging threats to their autonomy from extractive global superpowers [1–3] and the constraints of debt servicing [4], governments in LMICs must capitalise on this increased agency by boldly reshaping governance to prioritise investments in human development over single‑sector goals, in order to promote better outcomes across domains. We follow Sen’s definition of human development as a person’s freedom to live a life they have reason to value [5]. Prioritising human development means we must, in the words of Paul Farmer, ‘counter failures of imagination’ [6], to escape scarcity mindsets that prioritise quick fixes such as single-outcome sector-specific interventions over holistic interventions targeting multiple outcomes. We suggest this requires moving beyond both siloed priority setting and intersectoral collaborative approaches to governance. Instead, we must ask: what would governance look like if it were structured to support a holistic life‑course approach to human development?

This question stems from a wider body of work approaching human development from a holistic life‑course perspective to develop economic evaluation and financing tools, culminating in the development of a Human Development Framework for understanding investments across the life course [7]. Set against the literature on the impact of government siloes and barriers to collaborative governance, this debate raises the central question: what would governance look like if it were structured to support a holistic life‑course approach to human development?

Many social conditions that shape a person’s health also shape their education, wealth, and other outcomes. Yet, many government departments in the social sector operate as siloes, neither prioritising nor implementing investments that target common or overlapping causes to improve outcomes across sectors. These siloed approaches are sufficient in well-resourced contexts, where, for the most part, improving outcomes one by one is all that is needed to ensure maximum human development potential, as each sector can assume other outcomes are or will be addressed by another sector. However, to meaningfully improve people’s lives in under‑resourced contexts characterised by multiple obstacles, improving people’s chance of reaching their full human development potential requires a holistic life‑course perspective. For example, improved maternal and child nutrition supports better cognitive outcomes [8], but without opportunities for early learning these gains would not be realized as human development potential [9]. Prioritising human development requires reframing investments in the social sector as investments in human development.

We use investments in public health, the health care system serving a population, and the health of that population beyond the absence of disease as our example. Despite widespread recognition that health is shaped by social and biological determinants [10], in many countries the public health sector prioritises biomedical interventions before the social determinants of health (SDOH) [10–13]. When investing in the latter, they default to health-specific, single-outcome interventions that fail to account for the interconnected nature of the SDOH and the value of health for human development. While biomedical interventions are critical, they must be complemented by investments in the SDOH. Within a Human Development Framework, good health protects human development potential and contributes to the realisation and utilisation of that potential in later life, but it does not ensure it [7]. This conceptualisation of human development, following Sen, provides an expanded evaluation space that allows for the consideration of improvements across domains and the interactions between them.

A holistic life-course perspective on human development prioritizes interventions that consider the interactions between investments across domains, such as health, welfare, and education, over time [14]. For example, in adverse contexts, the benefits of early investments are often not sustained long-term, particularly for the most vulnerable [15]. Without subsequent investments, or ‘sustaining environments’ [16], early‑life gains may not only fade away [17] but may deepen existing inequalities, as people with access to later advantages benefit most from earlier investments [18]. In contrast, considering investments in multiple domains, at multiple time points likely leads to more equitable outcomes [7,15]. Recognising this should change how we invest in health. Instead of prioritising interventions that improve single health outcomes, we should target both the SDOH and, critically, the socio-economic circumstances that allow health improvements to be realised as human development.

Our central thesis is that if we make human development our goal, rather than individual social sector outcomes, including health, we need innovations in governance that move beyond both traditional sector-specific approaches and newer intersectoral frameworks towards restructured government models that fundamentally transcend sectoral boundaries. To illustrate this, we outline the problem with siloed government structures, the market-oriented logic underpinning these structures, and the limitations of intersectoral approaches to overcome these siloes. Considering the shortcomings of siloed and intersectoral models in prioritising human development, we explore alternative governance arrangements and their implications for priority setting in health.

We examine the limits of siloed approaches to human development in contexts of adversity, because the dangers of a narrow focus on health are greater and more frequent in contexts characterised by broad socio-economic and political constraints. As such, we are focused on LMICs, although we suggest the principles of our argument would hold for vulnerable populations in High-Income Countries (HICs).

## The limits of siloed and intersectoral approaches to governance

In many countries, government departments operate as siloes, each with independent aims, priority-setting mechanisms, and implementation strategies, limiting the uptake and implementation of programmes and policies that require cross-sectoral effort [19], such as food policy [20,21], mental health care [22,23], and early childhood development [24,25]. Their mandate incentivises each sector to invest primarily in addressing the proximal determinants of outcomes within their sector and undervalues interventions targeting common or overlapping causes of outcomes across sectors, despite the promotion of such ‘accelerator’ interventions by multilateral bodies like the United Nations Development Group (UNDG) and the evidence to support them [26]. For example, evidence suggests that in resource‑constrained settings a household cash transfer combined with parenting support simultaneously improves adolescent health, schooling, and safety outcomes, as these outcomes are all often shaped by household poverty [27].

The tendency to prioritize single-sector interventions is reinforced by sector-by-sector priority‑setting processes reliant upon narrow economic evaluations [28]. When economic evaluations are limited to the consideration of sector‑specific outcomes only, they can miss the value of benefits outside the implementing sector; thus, interventions which have lower single‑sector impact but greater cross‑sector impact will be undervalued and overlooked. Using the previous example of a ‘cash plus’ intervention: a sector-by-sector economic evaluation treats the cash transfer plus parenting support as less cost-effective than targeted health, education, or violence interventions, even though its combined effects across all three outcomes make it more efficient from a whole-of-government perspective [27]. When the full value of interventions with cross‑sectoral impacts is recognized, there are significant political and logistical barriers to their uptake.

Many countries subscribe to New Public Management (NPM), a market-based approach to public administration [29], which amplifies the pitfalls of siloed approaches to government. NPM attempts to overcome the inefficiencies associated with traditional bureaucracies by demanding government be run like private firms. Here, classical Weberian principles of hierarchical organisation, impersonal rules and procedures, and a clear division of labour are replaced with principles of decentralisation, departmentalisation, outsourcing, and performance management [30]. At the heart of NPM is ‘the creation of focused single-purpose [government] agencies driven by clear market-like incentives to perform ever better’ [31]. Its emphasis on managerial autonomy, decentralised responsibility, and narrow performance targets has created and reinforced policy silos [32] and established organisational structures that limit cross-sectoral collaboration [31]. Recognition of the limitations of siloed interventions has led to a welcome focus on intersectoral interventions to address common upstream causes and produce outcomes across multiple development domains [33,34].

Various government structures have been proposed and tested to overcome the bureaucratic and political resistance to intersectoral interventions, including whole‑of‑government approaches (WoG) [35], joined‑up government (JUG) [31], and, from a health perspective, population health management (PHM) [36]. However, while these approaches have had some success, they face challenges overcoming governance-related obstacles [37], political constraints in implementation [38], and are operationally time‑ and resource‑consuming [32]. Joined-up working, for instance, not only requires departments to agree to collaborate but also to share objectives, information flows, accountability arrangements and implementation processes across organisational boundaries that remain largely intact [31]. This is evident in the limited impact of South Africa’s Cluster System on improved interdepartmental coordination, despite its aim to promote interagency coordination and cross-cutting programmes in line with government‑wide priorities [39–41],

Globally, the impetus towards intersectoral approaches is evident in the Sustainable Development Goals (SDGs) [37] and Health in All Policies (HiAP). First implemented in Finland, HiAP makes health a shared governmental responsibility by bringing health and non-health sectors together in policy and strategy development and implementation [42–44]. Yet, like other forms of policy‑based intersectoral collaboration, HiAP implementation has at times been undermined by limited buy-in from non-health sectors, siloed organisational structures, competing priorities, weak cross-sectoral ownership and accountability, poor communication between stakeholders, and unstable political and organisational support [43–48]. These challenges are pronounced in LMICs, where weaker public institutions and greater bureaucratic fragmentation can make implementation especially difficult [49]. For example, in Kenya, HiAP has been formally incorporated into the 2014–2039 national Health Policy, yet intersectoral action remains ad hoc, awareness outside the health sector is limited, with little political ownership beyond the Ministry of Health [50].

HiAP aims to improve the SDOH, and while this is critical for health and can be expanded to pursue the co-benefits of health outcomes and health policies in other sectors [48], like other single‑issue‑focused approaches, it does not solve the challenge of fully realising and utilising the human development potential that good health protects. We suggest that human development, not health, be the guiding principle within the social sector. Prioritising human development requires approaching investments in health as investments in human development, rather than investments in the social sector as investments in health. This means both greater investments in the SDOH to realise their non-health benefits and investments in non-health interventions to realise health’s benefits for human development.

These examples illustrate how the primary strategy in intersectoral approaches has been to use political will to overcome bureaucratic resistance [32]. However, as discussed, political motivation for collaboration is often met with resistance from rigid bureaucratic structures, even when it is institutionalised. Like the marketized government structures shaped by NPM, many intersectoral initiatives are hamstrung by bureaucracy and effect few real changes in approach: ‘these grandiloquent strategies [have often] come down to issuing a new law or creating inter-ministerial committees, or to a handful of agencies acting in the name of a greater goal but without further implications in their daily bureaucratic routines’ [51]. This raises the question of what must happen for government to prioritise and implement investments that support a holistic life‑course approach to human development? We suggest the answer lies in structural changes to the social sector to remove sectoral boundaries.

## Governance for human development: what could we do?

In these times of poly-crises and political and economic uncertainty, maximising human development can prepare individuals and societies to face current and future challenges. We know what is at stake, and we know that fixes to governance reliant on political will while maintaining sectoral separation struggle to overcome the political, institutional and bureaucratic constraints within priority setting and implementation to set and meet common goals. While we cannot provide a complete solution here, we maintain that sectoral separation itself is a structural barrier that collaborative mechanisms operating within that separation cannot fully overcome. One way to ensure that investments that are implemented in one sector, say health, but benefit a different sector, say education, are not overlooked, is to unify the planning and priority‑setting process around a single goal, through structural changes to institutions. An example of this could be a Ministry of Human Development, replacing the Ministries of Health, Education, and Social Development. The Ministry of Human Development would lead policy making, priority-setting, and coordination of the Departments of Health, Education, and Social Development. These departments would then be responsible for the delivery of interventions, given their unique access to populations and specialised implementation expertise and delivery infrastructure. In this structure, delivery is specialised, but priorities are set at a higher level. This arrangement would incentivise the identification and implementation of interventions addressing common causes. In South Africa, a Ministry of Human Development could consolidate the Ministries of Social Development, Health, Basic Education, and, given the challenges in the provision of safe housing in South Africa and the impact of apartheid spatial segregation, the Ministry of Human Settlements. The Ministry of Human Development would thus replace the function of the Social Protection, Community and Human Development Cluster, allowing for joint welfare goals within a social sector united in action, not only in name.

Our approach builds on the partial precedent set by Finland’s joint Ministry of Social Affairs and Health but takes the level of integration further. Rather than asking sectors to incorporate health priorities, it makes health, education, and social development co-equal domains of priority-setting under an overarching human development mandate. Importantly, this approach is not synonymous with centralised national government. A Ministry of Human Development could be decentralized just as a Ministry of Health could. Key to the solution, we suggest, is not a concentration of political power at one level but a unification around a single goal without sectoral barriers. This approach differs from examples of human development-focused initiatives guiding aid, such as the United Kingdom’s Foreign, Commonwealth and Development Office’s (FCDO) Human Development Directorate [52], in that it would be motivated and implemented by a national government pursuing its own development aims.

The health system is easily caught in a binary debate between prioritising targeted health system interventions versus policies to address the social determinants of health, which neglects to consider the health system’s ‘stewardship function [which] includes strategic planning, design of clear and enforceable rules of the game, assessment of health system performance, and coordination within the health sector and with other sectors involved in the generation of goods and services with important health effects.’ [53] In our governance model, the health system’s stewardship role would be undertaken by the Ministry of Human Development, because it is not waylaid by the trappings of bureaucracy or political will in its directives to the Department of Health.

What we offer here is not the only solution, nor is it immune to the need for political will or without barriers; for example, there may be concerns regarding internal cohesion. Similarly, we cannot presume the full impact implementing such an approach may have in any context, given idiosyncrasies in governance systems. What we do know is that sectoral separations are a choice underpinned by normative, value‑based frameworks, rather than an intrinsic component of government. In choosing to prioritise human development, our values have shifted, and the shape of government can and should shift too.

## Conclusion

This debate aims to encourage government stakeholders to prioritise human development by adopting holistic interventions targeting common and overlapping causes of social ills and by supporting the gains from these interventions with subsequent investments, particularly for the most vulnerable. When considering how to make such interventions prioritised, this debate asks stakeholders to consider structural changes to governance by breaking down sectoral siloes rather than working across them. The proposed model is imperfect, and in its’ simplistic presentation here, is subject to valid criticisms. Yet it is critical that we explore alternatives to the governance status quo, even imperfect ones. Reshaping governance structures seeks to create a coherent, inclusive and sufficiently robust governance model that can respond to many constraints on our lives to give each of us the best chance of reaching our human development potential.
